# Pancytopenia and Severe Gastrointestinal Toxicities Associated with 5-Fluorouracil in a Patient with Thymidylate Synthase (TYMS) Polymorphism

**DOI:** 10.7759/cureus.798

**Published:** 2016-09-21

**Authors:** Bo Wang, Shannon J Walsh, Muhammad W Saif

**Affiliations:** 1 Internal Medicine, Tufts Medical Center, Tufts University School of Medicine; 2 Tufts Medical Center, Tufts University School of Medicine; 3 Hematology/Oncology, Tufts Medical Center, Tufts University School of Medicine

**Keywords:** 5-fu, tyms, rectal cancer, toxicity, mucositis, neutropenia, thrombocytopenia

## Abstract

5-Fluorouracil (5-FU) is one of the most commonly used chemotherapeutic agents in solid tumors, including colon, gastric and breast cancers. The pharmacogenetic syndrome of dihydropyrimidine dehydrogenase (DPD) deficiency leading to severe toxicity after administration of 5-flourouracil (5-FU) and capecitabine has been well-recognized. However, the data about the association of the target enzyme, thymidylate synthase (TYMS) with the toxicity of these agents is limited. A 50-year-old Caucasian woman with T2N2M0 Stage IIIB squamous cell rectal cancer after local surgical excision initiated 5-FU therapy with mitomycin-C and radiation therapy in the adjuvant setting. Following the first treatment with 5-FU, she developed grade III mucositis and grade IV neutropenia which delayed her second dose of therapy. Following her second dose of 5-FU, she again developed grade III mucositis, grade II diarrhea, pancytopenia, fever, and rectal bleeding requiring hospitalization. She was treated with blood and platelet transfusion, pegfilgrastim, IV antibiotics, and supportive therapy. Due to her severe clinical toxicity following chemotherapy involving 5-FU, we tested her for both DPD deficiency andTYMS polymorphisms. The patient was found to be homozygous for the TYMS polymorphism 5’TSER genotype 2R/2R*f, which has been associated with increased 5-FU drug sensitivity and susceptibility to 5-FU toxicity. Our case report further underlines the fact that TYMS polymorphism not only predicts response to 5-FU by relating to intratumoral-TYMS mRNA expression but also the toxicity in these patients receiving fluoropyrimidines. In brief, TYMS genotype variations present a dilemma in 5-FU-driven cancer therapy- overexpression leads to decreased drug sensitivity and poor prognosis, while underexpression leads to the manifestation of toxic drug effects that may halt therapy altogether. Future prospective translational studies in a larger population are warranted to validate its role as a predictive and prognostic factor.

## Introduction

5-Fluorouracil (5-FU) is an irreversible inhibitor of the enzyme thymidylate synthase (TYMS) and is the most frequently used drug in chemotherapy in a variety of gastrointestinal cancers [1]. Its action as an antimetabolite inherently harbors potential for toxic side effects, including high-grade diarrhea, stomatitis/mucositis, and neurotoxicity. Thymidylate synthase (TYMS) is responsible for the conversion of deoxyuridylate (dUMP) to deoxythymidylate (dTMP) as a requisite step toward DNA synthesis and is the primary inhibition target of 5-FU. Lower levels of tumor TYMS expression correlate well with improved response to fluoropyrimidines [2]. Individuals carrying the higher expression TYMS genotypes have significantly worse overall outcomes and progression-free survival in gastrointestinal cancers [3]. Patients who express polymorphisms with a greater quantity of 28 base-pair tandem repeats in the promoter region of the TYMS gene have a higher level of expression of TYMS mRNA and enzyme and have associated poorer response to 5-FU and less toxicity to the drug [4]. Conversely, patients harboring gene polymorphisms with fewer tandem repeats display an associated increased sensitivity to 5-FU therapy and a higher likelihood of adverse drug reactions. Another genetic variant, dihydropyrimidine dehydrogenase (DPD) deficiency is a familial syndrome secondary to allelic mutations in the DPYD gene. DPD deficient patients are prone to develop life-threatening toxicities from 5-FU therapy. One study of 615 patients showed that 80 patients (13%) suffered severe toxicities including five lethal events (0.8%) [4]. Of the patients with severe toxicity, it was found that DPD activity was impaired in 71% of patients and 80% in those showed a fatal outcome; drug levels up to 15 times higher than the non-toxic population were also observed.

Here we report a patient with TYMS 2R/2R (2 tandem repeats), homozygous polymorphic alleles receiving combination 5-FU and mitomycin-C (MMC) and radiation therapy (XRT) for Stage II rectal cancer who developed 5-FU associated toxicity and required hospitalization for severe neutropenia, thrombocytopenia, mucositis, and severe diarrhea. The institutional review board of Tufts Medical Center approved human subjects for the study. Informed consent was obtained for the study.

## Case presentation

A 50-year-old Caucasian female with a past medical history of hypothyroidism presented to her physician with a chief complaint of six months perianal rash and anal mass. Surgical excision of a 2.7 cm X 1 cm perianal mass demonstrated moderately differentiated invasive squamous cell carcinoma with basaloid features with positive margins and evidence of lymphovascular invasion. P16 immunostaining was diffusely and strongly positive.

She was referred to the cancer center for further workup. A computed tomography (CT) scan of the chest, abdomen, and pelvis with intravenous (IV) contrast demonstrated a small pericardial effusion, a 2 mm right upper lobe nodule and a 4 mm density in the right lower lobe suggestive of lymphoid aggregation. A magnetic resonance imaging (MRI) of the pelvis showed an ill-defined anal mass involving the left wall of the lower anal canal with the possible focal partial invasion of the adjacent external sphincter and adenopathy in the bilateral ischioanal fossae. A pedunculated lumbar uterine fibroid with cystic central degeneration was also found. The subsequent PET scans confirmed malignancy in the lower anal canal as well as in the left inguinal lymph node and endometrium without the involvement of perirectal nodes. However, the endometrial biopsy was negative for cancer.

She began treatment on Cycle 1 Day one with an MMC/5-FU/XRT regimen [5-FU 1000 mg/m^2^/day IV continuous infusion on Days one to four and 29-32 (maximum daily dose of 5-FU 2000 mg/day) plus MMC 10 mg/m^2^ IV bolus on Days 1 and 29 (maximum 20 mg per dose)] [5]. On Day 15 post therapy, she presented with grade 3 mucositis involving her tongue and cheek. Her absolute neutrophil count (ANC) was 400 k/uL (1.5-7.5 k/uL). Also, she was found to have a urinary tract infection. She was initiated on filgrastim 200 mcg/day for five days and Bactrim DS. After several days, she developed grade 2 rashes with boils secondary to radiation and grade 2 diarrhea with mucous and soft stool. Her ANC was 1000 k/uL. Complications involving grade 4 neutropenia and further severe mucositis delayed the next scheduled dose from one week up to Day 36.

**Figure 1:**
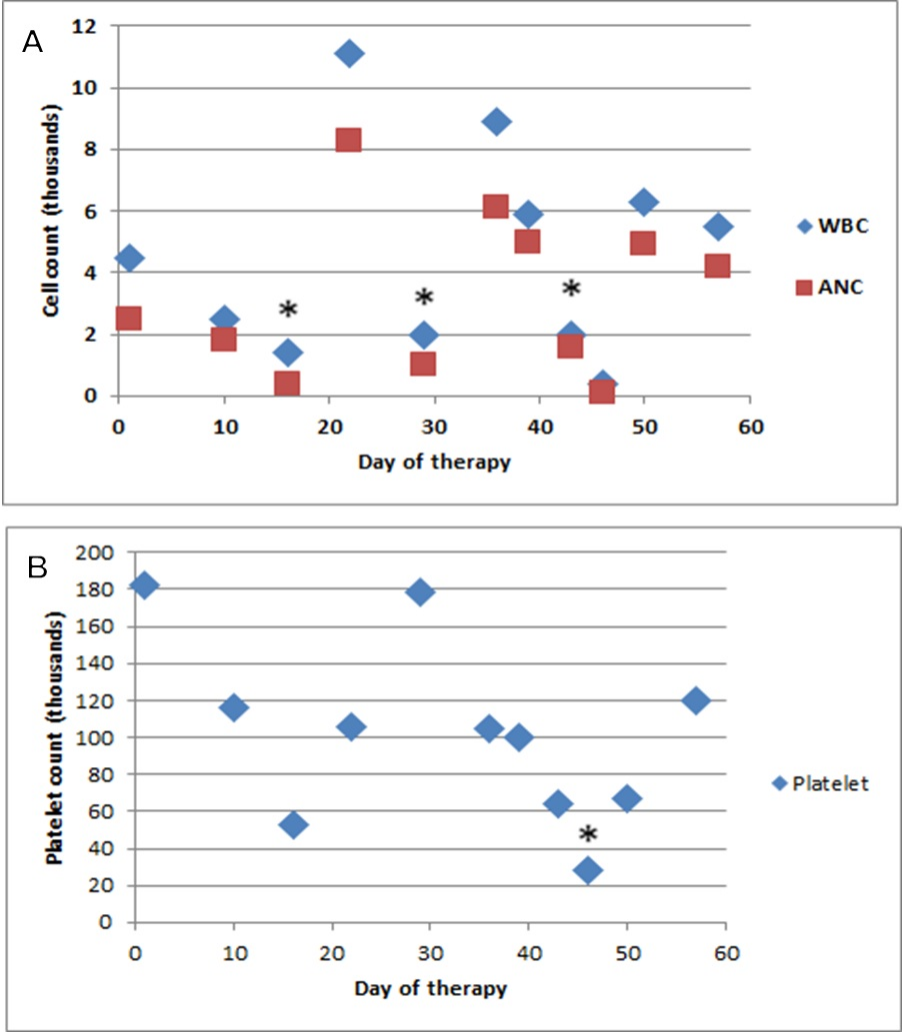
Leukocyte, ANC, and platelet count by day, after the initial administration of 5-FU. A. Leukocytes and ANC count by day, after the initial administration of 5-FU. A * indicates an intervention on the indicated day. B. Platelets by day, after the initial administration of 5-FU. A * indicates an intervention on the indicated day.

Within one week of dose two of chemoradiation, she received one dose of pegfilgrastim (Neulasta) for ANC of 1600 k/uL. This cycle was complicated by severe anorectal discomfort and grade 3 mucositis again. Three days following the pegfilgrastim administration, she developed subjective fevers, fatigue, rectal bleeding and was admitted with severe neutropenia and thrombocytopenia in addition to anemia. Her ANC was 100 k/uL, hemoglobin was 4.0 g/dL below her baseline of 12.2 g/dL, and platelets were 28,000 k/uL (150-400 k/uL) (Figures 1A-1B). The physical examination was significant for severe mucositis presenting as oral ulcers along lateral surfaces of her tongue bilaterally, perianal erythema with skin breakdown extending to the labia and a soft, non-tender mildly distended abdomen. Although afebrile, ciprofloxacin was started as prophylaxis. After developing a fever of 38.7^o^C, intravenous antibiotics were expanded to vancomycin (750mg every eight hours) and ertapenem (1 g every 24 hours). Serum cultures which were drawn before antibiotic administration all remained negative. The patient received silver sulfadiazine (one percent topical twice a day) and Aquaphor (topical twice a day) for perianal mucositis. Her diarrhea resolved on Day three of hospitalization. An abdominal CT showed no evidence of colitis. Following a reaction from the initial platelet transfusion involving hives and periorbital edema, she was successfully transfused one unit of packed red blood cells and two units of platelets with a pre-infusion protocol. Her neutropenia resolved on Day four of hospitalization with ANC of 2.6 k/uL and she remained afebrile until her discharge on Day five from admission.

Her rapid onset of complications including severe neutropenia and dequantization prompted us to test for genetic mutations before administration of dose two of her regimen. However, the results were not available until following the delivery of dose two. Pharmacogenetic tests of the DPD gene and TYMS gene were performed. Dihydropyrimidine Dehydrogenase (DPD) Gene Mutation Analysis (Quest Diagnostics Nichols Institute-San Juan Capistrano, CA), was negative for the IVS14+1G>A mutation in the DPD gene, which accounts for 50% of the DPD deficiency alleles. The 5-Fluorouracil Toxicity and Chemotherapeutic Response Panel (Quest Diagnostics/Nichols Institute – Chantilly, VA), indicated two mutations (2R) of a 28 base-pair tandem repeat in the 5’ promoter enhancer region (5’-TSER) on both alleles (2R/2R) of the thymidylate synthase (TYMS) gene. This homozygous genotype predicts low TYMS expression, increased 5-FU responsiveness, and an increased risk of toxicity [6]. The six base-pair deletion variant rs16430 in the 3’-untranslated region (UTR) of the TYMS gene was not detected. The commonly found DPYD gene mutations (1679 T>G, 1905+1 G>A, 2846 A>T) were not detected either.

## Discussion

This report documents a female patient with stage IIIb rectal squamous cell carcinoma receiving definitive chemoradiation therapy with 5-FU/mitomycin/XRT who developed severe toxicity to 5-FU secondary to a 2R/2R polymorphism in the TYMS gene. The common toxicities to 5-FU include cytopenia, diarrhea, mucositis, alopecia, and neurotoxicity. In this case, the patient experienced profound pancytopenia (particularly neutropenia and thrombocytopenia), high-grade diarrhea, and mucositis throughout the gastrointestinal (GI) tract. Her polymorphism predisposed her to a higher 5-FU drug sensitivity through an inherently decreased basal level of intracellular TYMS mRNA and enzyme. The 2R/2R variation of TYMS is associated with a ≤ 2.5-fold risk of toxicity to 5-FU/capecitabine. This elevated risk of toxicity is due to a lower level of enzyme in normal tissue cells which means that 5-FU cytotoxic characteristics more easily harm benign cells, but 5-FU more effectively acts as an antimetabolite in tumor cells due to lower levels of intratumoral TYMS mRNA. An alternate TYMS mutation, not found in our patient, manifests as a six base-pair (bp) variation in the 3’-untranslated region of TYMS. A six bp deletion at bp 1494 leads to lower levels of TYMS expression in colorectal tumors, an increased 5-FU sensitivity, and a risk of toxicity whereas a 6bp insertion lead to higher levels of TYMS and poorer prognosis [7]. A relatively recent meta-analysis based on results from the QUASAR 2 study concluded that variants TYMS 5VNTR 2R/3R (rs45445694) and TYMS 3UTR 6bp ins-del (rs16430/rs151264360) were found to display significant association with global grade 3+ fluoropyrimidine toxicities in Caucasian patients who received capecitabine adjuvant chemotherapy for stage II/III colorectal carcinoma [8].

Other genotype variants have been associated with highly limiting toxic side effects of 5-FU as well. For example, mutations in DPYD, the gene encoding DPD enzyme, lead to poor drug clearance of 5-FU metabolites. DPD is normally responsible for 80% of 5-FU metabolism. When mutations leading to decreased DPD enzyme activity or production occur, 5-FU accumulates, leading to systemically toxic effects similar to or more severe even than those seen in individuals with TYMS polymorphisms. In their meta-analysis, Rosmarin et al., demonstrated significant DPYD c.2846 TA (rs67376798) association with global toxicity from capecitabine therapy [8]. Interestingly, DPYD*2A (also known as 1905+1 G>A) was not significantly associated with predictable toxicity despite previous reports of association with 5-FU toxicity in prior literature [9], suggesting that further studies must be performed to develop reliable assays to predict for 5-FU toxicity before therapy is initiated.

The population frequency of TYMS variants associated with 5-FU toxicity is not known among the general population as well as different ethnicities. However, given our data in this case and other reports by our group as well as by other investigators worldwide, we suggest that larger randomized studies should test the role of TYMS and DPD screening before 5-FU therapy in cancer patients to prevent untoward toxicity as more agents are available these days. Also, the pharmacokinetic module should decide the dose that may work in complement with these genetic markers in defining the dose before administration of these chemotherapy drugs [10].   

## Conclusions

TYMS genotype variations present a dilemma in 5-FU driven cancer therapy - overexpression leads to decreased drug sensitivity and poor prognosis while underexpression leads to the manifestation of toxic drug effects that may halt therapy altogether. However, given our data in this case and other reports by our group as well as by other investigators worldwide, we suggest that randomized studies should test the role of TYMS and DPD screening before 5-FU therapy in cancer patients to prevent an untoward toxicity, as more agents are available.
